# Spatial and temporal functional changes in alpine summit vegetation are driven by increases in shrubs and graminoids

**DOI:** 10.1093/aobpla/plu008

**Published:** 2014-02-21

**Authors:** Susanna Venn, Catherine Pickering, Ken Green

**Affiliations:** 1 School of Environment, Environmental Futures Centre, Griffith University, Gold Coast, QLD 4222, Australia; 2 Department of Botany, Research Centre for Applied Alpine Ecology, La Trobe University, Bundoora, VIC 3086, Australia; 3 New South Wales National Parks and Wildlife Service, Snowy Mountains Region, PO Box 2228, Jindabyne, NSW 2627, Australia

**Keywords:** Alpine vegetation, community composition, functional composition, functional traits, GLORIA, Snowy Mountains.

## Abstract

Classical approaches to investigating temporal and spatial changes in community composition offer only partial insight into the ecology that drives species distribution, community patterns and processes, whereas a functional approach can help to determine many of the underlying mechanisms that drive such patterns. Here, we aim to bring these two approaches together to understand such drivers, using an elevation gradient of sites, a repeat species survey and species functional traits. We used data from a repeat vegetation survey on five alpine summits and measured plant height, leaf area, leaf dry matter content and specific leaf area (SLA) for every species recorded in the surveys. We combined species abundances with trait values to produce a community trait-weighted mean (CTWM) for each trait, and then combined survey results with the CTWMs. Across the gradient of summits, more favourable conditions for plant growth (warmer, longer growing season) occurred at the lower elevations. Vegetation composition changes between 2004 and 2011 (according to non-metric multi-dimensional scaling ordination) were strongly affected by the high and increasing abundance of species with high SLA at high elevations. Species life-form categories strongly affected compositional changes and functional composition, with increasing dominance of tall shrubs and graminoids at the lower-elevation summits, and an overall increase in graminoids across the gradient. The CTWM for plant height and leaf dry matter content significantly decreased with elevation, whereas for leaf area and SLA it significantly increased. The significant relationships between CTWM and elevation may suggest specific ecological processes, namely plant competition and local productivity, influencing vegetation preferentially across the elevation gradient, with the dominance of shrubs and graminoids driving the patterns in the CTWMs.

## Abstract (precis)

In order to determine the mechanisms that drive changes in plant community composition across spatial and temporal scales, plant functional traits were used to interpret the results of a repeat species survey across a gradient of five alpine summits in south-east Australia. Vegetation changes were strongly affected by the high and increasing proportion of tall shrubs and graminoids, especially at the lower elevation summits. Several significant relationships between the community trait-weighted mean of different traits and elevation may suggest processes such as competition are influencing vegetation preferentially across the elevation gradient, with shrubs and graminoids driving these patterns.

## Introduction

In the context of environmental change, linking quantitative measures of plant species physical characteristics, for example their functional traits or life form, with species distributions and local environmental factors can reveal the processes that drive patterns in vegetation composition (Díaz and Cabido 1997; Tilman *et al.* 2001; Lavorel and Garnier 2002; McGill *et al.* 2006). The interaction among functional groups of species with climatic changes undoubtedly affects community composition and underlying ecosystem functioning (Hooper and Vitousek 1997; Westoby and Wright 2006; Spasojevic and Suding 2012). Hence, measures of functional traits, functional diversity or functional composition, in combination with measures of species composition and diversity, may provide greater insights into the assembly processes that drive community composition and for gauging ecosystem stability in changing environments (Tilman *et al.* 1997).

Using environmental gradients is a simple way to examine natural variation in vegetation and community responses to environmental changes (McGill *et al.* 2006). Intraspecific variation and species turnover can cause shifts in trait values differentially across gradients, and thus functional diversity across environmental gradients is a function of biotic and abiotic interactions (Venn *et al.* 2011; Schöb *et al.* 2012; Spasojevic and Suding 2012), disturbance regimes (Choler 2005), population dynamics (Pollock *et al.* 2012) and biogeochemical cycling (Mason *et al.* 2012) at local, regional and biome scales (Reich *et al.* 1997). Mountainous regions are therefore ideal for testing the role of plant functional traits across environmental gradients in determining community composition and shifts therein; over relatively short spatial scales, rapid changes in elevation interact with local topography to create steep gradients in temperature and precipitation (Körner 1999). Thus, the variation in species abundance across an environmental or elevation gradient will impact on community composition and ecosystem functioning (Tilman *et al.* 1997), as different processes are affected by different species via changes in the representation of species functional traits (Chapin *et al.* 1996). The interrelation between a species life form and its functional traits is exemplified across elevation gradients in alpine regions; for example, shrubs (taller, long-lived, woody plants) dominate in favourable environments. Through their morphological and physiological traits, shrub species can modify a wide range of ecosystem processes, including alteration of local snow depths and associated hydrological dynamics, nutrient exchange and associated net carbon balance (Myers-Smith *et al.* 2011). In addition, shrub species are often taller than neighbouring forbs and they can be competitively superior, forming dense thickets with closed canopies. Increases in shrub cover and height can also potentially restrict the growth of other plant species by limiting light availability (Myers-Smith *et al.* 2011). Graminoids, namely grasses and sedges, however, may compete with shrubs in the more favourable environments, recruit within the canopy of senescing shrubs (Williams and Ashton 1988) and are well adapted to a range of environmental conditions; many C3 grasses and sedges can also rapidly increase in abundance after sufficient rainfall and improved abiotic conditions (Jarrad *et al.* 2009).

Here, we use community trait-weighted means (CTWMs) to explore the driving mechanisms of temporal change in vegetation composition across an elevation gradient of alpine summits. We used four easily measured morphological traits: plant height at maturity, leaf area, leaf dry matter content (LDMC) and specific leaf area (SLA); some of these are interrelated, but all represent important dimensions of functional and strategic variation among plant species (Westoby 1998; Weiher *et al.* 1999) and are responsible for some of the most striking and important patterns in species distributions in the field (Westoby *et al.* 2002; Choler 2005). In addition, large-scale comparisons among biomes provide evidence that these traits may be viewed as relevant functional markers suitable for predicting species performance along gradients (Reich *et al.* 1997). While we did not measure community assembly processes directly, these chosen traits are important for various assembly processes and population dynamics such as competition, facilitation, productivity, and stress tolerance and longevity (Grime 1977; Westoby 1998; Cornelissen *et al.* 2003; Schöb *et al.* 2012; Michalet *et al.* 2014). We can therefore infer the mechanisms behind changes in community composition based on life forms, individual traits and patterns in the CTWMs. The elevation gradient of sites represents a strong gradient of temperatures, snowmelt date and growing season length, and therefore represents an important gradient across which community composition and functional composition are expected to vary preferentially, according to position along the gradient. Specifically we ask: what is the spatial and temporal variation in species composition across the gradient of sites; and where are various combinations of traits and life forms most prevalent? We then discuss how these composition patterns and functional traits interact with species' ecology preferentially across the gradient of sites.

## Methods

### Study sites

**Figure 1.**
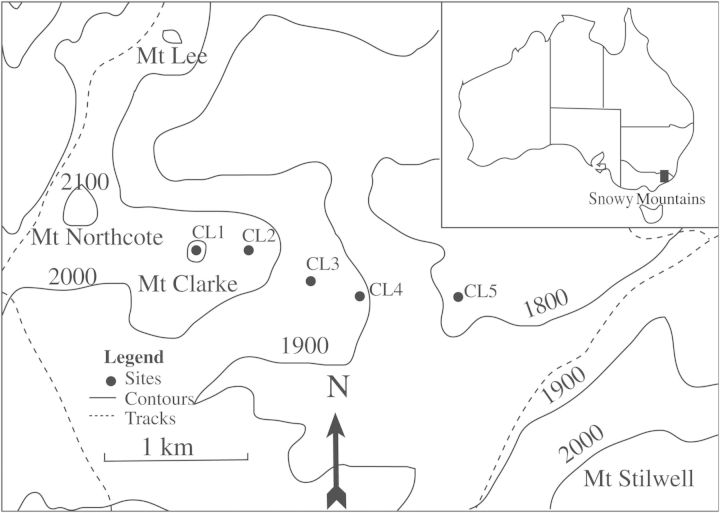
Location of the Snowy Mountains in Australia and study site locations at Mt Clarke 1–5 (CL1, CL2, CL3, CL4, CL5) representing the five summits.

We used five alpine summits in this study that represent the Australian contribution to the Global Observation Research Initiative in Alpine Environments (GLORIA): an ongoing, empirical study with the specific aim of detecting alpine vegetation change on summits in relation to climate change. In January 2011, we re-surveyed the summits that were originally marked and surveyed in January 2004 that exist along a continuous ridge from close to the valley floor to the summit of Mt Clarke (Pickering *et al.* 2008; Pickering and Green 2009) (Fig. 1). The summits cover an elevation range of 301 m from the lowest at 1813 m (Clarke 5) through to the highest at 2114 m (Clarke 1) (Table 1) and cover a horizontal distance of 1600 m. The summits were selected for long-term monitoring under the GLORIA sampling protocols (Pauli *et al.* 2004), as they experience similar effects of exposure and differences in climate that are most likely due to the elevation gradient. They are all relatively flat, rather than cone-shaped peaks, and the vegetation is characteristic of nearby summits. The soils are around 350 ± 110 mm in depth (K. Green, unpubl. res.), well-formed alpine humus soils (Costin 1954). The highest summits are dominated by tall alpine herbfield (a mixture of forbs and graminoids), whereas the lower summits are characterized by shrubs. As a result of the continuous, mostly perennial vegetation cover, biomass is high compared with some other alpine regions (Costin 1954). There are some rock outcrops, but these are not a defining feature of the summits. Disturbance is minimal as cattle grazing ceased >60 years ago, the historical stock travelling route avoided these summits and there are few native and no exotic burrowing mammals at these elevations. No walking tracks cross the summits, resulting in low visitation rates.

**Table 1.** Summit details including locations, elevation (metres above sea level (m.a.s.l.)) and area (m^2^); the mean species richness and overlapping vegetation cover for each of the upper and lower SASs divided into three life-form groups (graminoids, forbs and shrubs) for 2011 and the change in mean species richness since 2004 (increase + or decrease −) at each summit; and the CTWM for four traits (height, leaf area, LDMC and SLA) for each of the sampling units for 2011 and the change in overlapping cover (%) since 2004 at each summit.

	Clarke 1		Clarke 2		Clarke 3		Clarke 4		Clarke 5	
Location	E 148.2875, S 36.4328		E 148.2911, S 36.4328		E 148.2961, S 36.4347		E 148.3000, S 36.4356		E 148.3078, S 36.4356	
Elevation (m.a.s.l.)	2114		2079		1992		1948		1813	
5 m SAS area (m^2^)	4722		3373		2860		3435		2212	
10 m SAS area (m^2^)	7581		8196		8551		8920		8664	
Mean species richness per area	2011	Change +/−	2011	Change +/−	2011	Change +/−	2011	Change +/−	2011	Change +/−
Upper mountain SAS (mean of aspects)	21.2	−0.5	23	+0.3	31.8	+0.5	31	+2.3	30.3	−1.3
Lower mountain SAS (mean of aspects)	25	+4	28.5	+2.8	34.5	+4	34.7	+11.5	32.3	+4.5
Vegetation (overlapping cover %)	2011	Change (%)	2011	Change (%)	2011	Change (%)	2011	Change (%)	2011	Change (%)
Upper mountain SAS (sum for summit)										
Graminoids	72.6	+22.4	50.9	+30.4	82.6	+90.7	67.3	+115	79.1	+324.7
Forbs	25.6	−5.0	14.2	+10.2	13.7	−33.8	18.9	+11.3	12.3	+85
Shrubs	9.5	+29.1	42.6	+23.9	14.2	+12.5	50.5	+34	92.7	+38.4
Lower mountain SAS (sum for summit)										
Graminoids	62.0	+25.7	68.6	+46.3	81.1	+75.4	63.1	+96.1	81.6	+150.3
Forbs	34.3	+10.8	25.5	−17.6	18.4	−26.3	28.8	+46.5	22.2	+349.4
Shrubs	18.1	+18.1	30.4	+53.5	14.6	−8.0	47.7	+12.6	65.4	+12.7
Community trait-weighted mean	2011	Change (%)	2011	Change (%)	2011	Change (%)	2011	Change (%)	2011	Change (%)
Upper mountain SAS (mean of aspects)										
Height (mm)	128.0	−3.3	116.3	+4.5	154.1	−10.1	180.9	−5.1	227.5	−21.2
Leaf area (mm^2^)	396.2	−8.9	237.5	+3.7	291.7	−1.6	208.7	+10.3	138.3	+104.4
LDMC (mg g^−1^)	192.0	+4.8	299.5	−7.5	183.3	−14.3	335.2	−7.8	382.3	−16.1
SLA (mm^2^ mg^−1^)	38.4	+6.8	30.2	+4.7	40.6	+21.6	28.5	+19.6	20.6	+92.8
Lower mountain SAS (mean of aspects)										
Height (mm)	121.6	+0.5	123.9	+0.8	144.6	−6.7	187.9	−12.9	226.5	−27.4
Leaf area (mm^2^)	354.9	+2.5	297.5	−15.9	307.1	+13.7	258.3	+16.1	163.4	+110.3
LDMC (mg g^−1^)	225.2	−3.9	248.7	+6.3	184.3	−18.8	317.7	−6.2	346.8	−16.6
SLA (mm^2^ mg^−1^)	32.5	+6.8	35.2	+14.7	41.1	+26.8	28.0	+18.4	22.2	+84.6

### Variation in abiotic variables across the gradient of summits

The variation in soil temperatures, nutrients and minerals across the elevation gradient has been described previously for the summits (Pickering *et al.* 2008; Pickering and Green 2009; Venn *et al.* 2012*a*). Temperature recording began at the summits in January 2002 using temperature loggers (Tinytag Plus—Gemini Data Loggers, Chichester, UK) buried 10 cm below the ground surface. From January 2004 to the present, loggers have been in position at the ‘corner’ of each aspect of each summit. Temperatures were recorded every 2 h. Temperature data from 2004 to 2011 were used to calculate annual values of absolute minimum soil temperature, annual daily mean soil temperature, absolute maximum soil temperature, temperature sums (>5 °C), growing degree days and the length of the growing season across the years sampled. Several climate parameters were derived from these data and used in previously published analyses (Pickering and Green 2009; Venn *et al.* 2012*a*).

Precipitation data were collected from 2003 to 2011 from a Bureau of Meteorology automated weather station about 8 km to the south at Thredbo (1957 m), and from Pengilley Bog (1730 m, during the growing season only), 13 km to the north-east.

Soil samples were collected in 2003 at each summit, on each aspect close to the 1-m^2^ quadrat clusters, giving a total of four individual soil samples per summit. A minimum of 500 g was collected in a single sample, taking the top 10 cm of soil through a 75-mm core. Samples were air-dried, sieved through a 2-mm mesh and analysed by the CSIRO Division of Soil and Water, Canberra, Australia. Samples were analysed for 17 minerals/nutrients (see Pickering and Green 2009 for full details).

### Vegetation sampling

The top section of each summit was divided into eight summit area sections (SASs), four covering the area down to 5 m below the summit, the 5-m isoline, for each of the four cardinal compass bearings (hereafter referred to as the upper or 5-m SAS), and another four covering the four compass bearings down to the 10-m isoline (hereafter referred to as the lower or 10-m SAS) (Pauli *et al.* 2011). Where the summit was exceptionally flat, the upper sampling area extended 50 m from the summit and the lower area extended 100 m. In each of the eight SASs the percentage cover of each vascular plant species was estimated using a random step-pointing method (200 points per area). In this method, vegetation is sampled at randomly generated points across the site, and hits are summed for each species in order to provide a measure of species abundance. Any species not sampled by the step-point method were visually assessed for cover (Pauli *et al.* 2011).

Sampling in 2011 was conducted ‘blind’ without referring to the 2004 data, and was performed by the same people to ensure consistency in the data over the days of fieldwork (Vittoz *et al.* 2010). Postsampling, a rigorous species identification checking procedure, was used to ensure that changes in species between 2004 and 2011 were accurate. All species names follow Costin *et al.* (2000) to be consistent with the initial 2004 survey.

We selected four important, character-based plant traits (plant height, leaf area, LDMC and SLA) (Table 2) to use in the functional composition analyses. Trait data, including destructive sampling, were not directly measured from plants within the study sites; rather all traits were collected from adjacent areas <200 m from the study sites, from within the Kosciuszko National Park alpine area in growing seasons of 2010, 2011 and 2012, using 10 adult individuals for each measurement. We chose these traits based on the individual plant and ecosystem functions in which they are involved (Lepš *et al.* 2006; Petchey and Gaston 2006), in the context of alpine plant community dynamics (Table 2).

**Table 2.** Plant traits measured for all species recorded in the upper and lower SASs. Traits were measured in the field and laboratory based on protocols outlined by Cornelissen *et al.* (2003).

Trait (unit)	Description	Functional indicator
Plant height (mm)	Shortest distance between the upper boundary of the main photosynthetic material (usually the canopy) and ground level	A measure of species overall competitive ability at plant maturity. Species that are relatively taller will be more competitive, usually for light. Indirect measurement for biomass, lateral spread, rooting depth and leaf size
Leaf area (mm^2^)	One-sided projected surface area of an average leaf	A measure of stress tolerance. Small leaves tend to be favoured under heat stress, cold stress, drought stress and high-radiation stress. Within a climate zone, leaf size tends to increase with plant height and soil nutrients, but decreases with disturbance. Larger leaves are expected in more productive landscapes
LDMC (mg g^−1^)	The ratio of the oven-dry mass of a leaf to the fresh weight of the leaf	Indirectly represents the mean density of leaf tissue, relates to the inverse of SLA. Low LDMC can indicate fast resource acquisition. Leaves tend to be more resistant to physical stress such as wind and hail. Species with low LDMC tend to be associated with highly disturbed environments and high productivity
SLA (mm^2^ mg^−1^)	Ratio of one-sided area of a fresh leaf to its oven-dry mass	Low values correspond to relatively high investments in defences to harsh conditions including long life spans and structural adaptations. Reflects the expected return on previously captured resources such as light and nutrients. A good positive correlate of potential growth rate

### Data analysis

#### Vegetation composition

In order to determine shifts in species composition and abundance between 2004 and 2011 across the gradient of sites, we used non-metric multi-dimensional scaling (NMDS) and ordination. Species abundance data from the upper and lower SASs were log + 1 transformed to down-weight very common species, and dissimilarities between all pairs of aspects from the upper and lower SASs were calculated using the Bray–Curtis dissimilarity coefficient (after 999 iterations), previously noted as a robust measure in recovering ecological distance over a range of models and stochastic variations in the data (Bray and Curtis 1957; Quinn and Keough 2003). How good the dissimilarity matrix is was determined by the stress value (Kruskal 1964; McCune and Mefford 1999). Stress values <0.2 are recommended (Clarke 1993) as values above this threshold may mislead interpretations.

The difference in vegetation composition between 2004 and 2011 was analysed by an analysis of similarities (ANOSIM) procedure (Clarke 1993; Quinn and Keough 2003). This procedure is analogous to an analysis of variance comparing between-group and within-group variation. The ANOSIM procedure tests the null hypothesis that there are no differences between *a priori* defined groups (in this case years), or that the average rank of dissimilarities between all possible pairs of objects in different groups is the same as the average rank of dissimilarities between pairs of objects in the same groups (Quinn and Keough 2003). All ANOSIM procedures for abundance data from the three spatial scales used permutation/randomization methods on a similarity matrix to allocate objects randomly to groups and then generate the distribution of *R* under the null hypothesis that all random allocations are equally likely (Clarke 1993; Quinn and Keough 2003). The *R* distribution is scaled between pairs of objects in the same group with values between −1 and 1. Differences between groups would be suggested by *R* values greater than zero where objects are more dissimilar between groups than within groups. *R* values of zero indicate that the null hypothesis is true. *R* = 1 indicates that all samples within groups are more similar to each other than to those in different groups (Quinn and Keough 2003).

The dominant character species, those that are useful in discriminating between years at each of the spatial scales, were explored using the similarity percentages (SIMPER) procedure (Clarke 1993), performed using the PRIMER 6 package (Plymouth Routines in Multivariate Ecological Research 5.1.2. 2010). This procedure utilizes the similarity and dissimilarity indices between all pairs of samples to identify typical species within a site/year, as well as important species that distinguish between years.

#### Community trait-weighted means

In order to illustrate functional changes between 2004 and 2011 across the gradient of summits, we calculated the CTWM for the assemblages of plants recorded in the upper and lower SASs at every summit, for each of the four traits. The CTWMs were calculated by the method proposed by Mason *et al.* (2003), interpreted by Mason *et al.* (2005) and modified by Lepš *et al.* (2006). This method allowed us to calculate the relative abundance of individual trait responses to the gradient of sites and over time, weighted by the absolute individual species abundances, rather than provide an aggregate index based on multi-trait space (Villeger *et al.* 2008). We utilized the software of Lepš and de Bello (2008) to assist in scaling our trait and abundance values for the data from the three spatial scales and to calculate the CTWM values.

Temporal change across the summits in CTWM was measured as the per cent difference between mean values between 2004 and 2011, and by examining the overlap of 95 % confidence intervals from a normal distribution of the data across the three spatial scales (Cumming and Finch 2005). Combining all sites, the difference between the 2004 and 2011 values of functional composition for each trait was compared with paired *t*-tests using the quadrants of each site (aspects) as replicates. The relationships between site elevation and the abundance-weighted trait means were assessed with simple linear regression. Additionally, the relationships between the vegetation composition across sites and trends in the CTWMs at those sites were illustrated by overlaying vectors on each of the two-dimensional ordinations, for both the upper and lower SASs, in order to illustrate which traits are more abundant and display any trends across the ordination, as well as their direction and influence in relation to the compositional floristic data. Only the community-weighted trait means were used as vectors, and only those with correlations to each ordination where *R*^2^ > 0.5 are displayed.

Ordinations, ANOSIM and SIMPER routines were performed using the PRIMER 6 package (Plymouth Routines in Multivariate Ecological Research 2010). SYSTAT ver. 10 (Copyright SPSS Inc., 2000) was used for all other statistical analyses.

## Results

### Variation in abiotic variables across the elevation gradient

Across the gradient of sites, soil temperature and growing season length have been shown in previous analyses to vary predictably with elevation. Repeatedly, more favourable conditions for plant growth have been found at the lower-elevation summits, with cooler, shorter growing seasons with (often) less available nutrients at the higher summits (Pickering and Green 2009; Venn *et al.* 2012*a*). As previously described in Venn *et al.* (2012*a*), there were no consistent summit-specific trends (increases or decreases) in climatic variables across the summits between 2004 and 2011, although relationships between summit elevation and climate between 2004 and 2011 remained consistent; minimum temperatures (linear regression: *R*^2^ = 0.28, *P* = 0.016), mean temperatures (linear regression: *R*^2^ = 0.44, *P* = 0.005) and number of growing season (snow-free) days (linear regression: *R*^2^ = 0.23, *P* = 0.032) all significantly decreased with increasing elevation but not maximum temperatures (using the data from four temperature loggers at each site as replicates) (Venn *et al.* 2012*a*).

Although precipitation was not specifically measured at each summit, data from the nearby rain gauges revealed substantial increases in annual and growing season precipitation over the 2010/2011 growing season (October to April) in comparison with previous years in which the region experienced low rainfall conditions for almost a decade (Venn *et al.* 2012*a*, *b*). At Thredbo, there was a 30 % increase in annual precipitation in 2011 compared with 2004 (mean precipitation between 2004 and 2009 was 1136 mm, whereas in 2011 it was 1647 mm). Growing season precipitation at Pengilley Bog was about 50 % higher in 2011 compared with previous years (mean growing season precipitation between 2003/2004 and 2009/2010 was 575 mm, whereas in 2010/2011 it was 1182 mm) (Venn *et al.* 2012*a*).

Soil nutrient analyses conducted in 2003 revealed few trends across the elevational gradient of sites (Pickering and Green 2009). Importantly, however, organic carbon (%), available nitrogen (as ammonium) and aluminium (mg kg^−1^) all decreased with increasing elevation.

### Temporal change in vegetation composition

Overall, species richness between 2004 and 2011 tended to increase at all sites (Table 1), with the largest mean increase occurring in the lower mountain SAS at Clarke 4 (+11.5 species). Vegetation composition changes between 2004 and 2011 across the gradient of sites were most pronounced among graminoids and forbs at the lowest site, Clarke 5, with an increase in forb abundance of up to 350 % in the lower mountain SAS (Table 1). Notable increases in graminoids were partly made up of the presence of new species in 2011 in many SASs across all summits, namely *Agrostis* sp., *Deyeuxia crassilisica*, *Australopyrum velutinum* (Poaceae), *Carex breviculmis* (Cyperaceae) and *Luzula alpestris* (Juncaceae).

Changes in abundance across life forms were generally within the same order of magnitude between the upper and lower SASs on each summit. Shrub abundance increased substantially in the lower SASs at the two highest sites, Clarke 1 and 2 (Table 1). Simple linear regression revealed few discernible patterns in the change in abundance of particular life forms between years across the gradient of sites, except for within graminoids in the upper and lower SASs, with per cent change decreasing significantly with site elevation (upper SAS: *R*^2^ = 0.92, *P* = 0.008; lower SAS: *R*^2^ = 0.99, *P* = <0.001).

**Figure 2.**
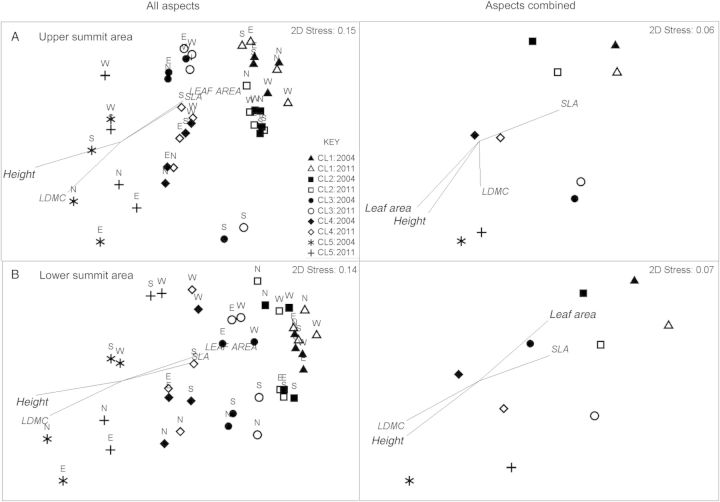
Non-metric multi-dimensional scaling ordinations of the (A) upper and (B) lower SASs at two levels of replication, within the five Mt Clarke summits using aspects, north, east, south, west, as replicates, and whole summits, to illustrate differences in vegetation composition (abundance) between 2004 (closed shapes) and 2011 (open shapes). Sites (shapes) close to each other in ordination space indicate high levels of similarity in species composition. Vector lines represent the CTWM values for the plant traits (height, leaf area, LDMC, SLA) with a Pearson's correlation of >50 % with the vegetation data for each year and sampling unit. Vector lines indicate the degree to which those functional traits affect the groupings (similarity) of sites in ordination space.

Compositional changes between 2004 and 2011, illustrated by the ordination diagrams, point to similar directional changes across all summits and sampling areas. In general, the vegetation composition in 2011 has changed in a consistent manner within and among sites in ordination space (Fig. 2), tending towards groupings of species with similarly high SLA. This also indicates that the turnover and abundances of key species have been similar across sites. This is most pronounced in the diagram illustrating the combined summit data for the lower SASs, indicating that the vegetation composition between 2004 and 2011 changed similarly across summits (Fig. 2B). The ANOSIM results point to significant differences in the vegetation composition between sample times, using aspects as replicates across sites (Table 3) within quadrats, the upper and lower summit areas.

**Table 3.** Results from the ANOSIM procedure across summits at the three sampling units and two levels of replication within sites (summits), comparing the similarity in vegetation data between 2004 and 2011.

Sampling unit/replication	*n*	Global *R*	*P*
Upper SAS/summit aspects	10	0.04	0.01
Upper SAS/summits	5	0.09	0.19
Lower SAS/summit aspects	10	0.07	0.05
Lower SAS/summits	5	0.06	0.25

The vectors that represent the functional composition in the community-weighted trait means, overlaid on the ordination diagrams, point clearly to the higher-elevation summits having high abundances of species with higher SLA ratios and larger leaf areas (Fig. 2). Conversely, the lower-elevation summits appear to have proportionally higher abundances of species that are taller and have larger LDMC values. Graminoid and shrub abundance tended to contribute to the majority of the differences between 2004 and 2011, revealed through the SIMPER analyses, using aspects on summits as replicates and with the data combined for each summit (Table 4). The abundances of the top 10 most influential and discerning species generally increased between sample times (Table 4), leading to the dissimilarity in vegetation composition between 2004 and 2011 as identified in the ANOSIM analyses. At the lower and higher SASs across sites, shrub species were identified as the most influential in determining differences between years (at least 5/10 of the top species were shrubs, using aspects as replicates and with data combined for each summit respectively) (Table 4). Graminoid species were the second most influential group leading to dissimilarity between years.

**Table 4.** The mean overlapping cover (%) in 2004 and 2011 of the 10 most typical species within each sampling unit and level of replication that contribute to the differences seen in the ordinations and ANOSIM analyses and the contribution (%) of each species to the total species pool in each group, using the SIMPER routine (Primer ver. 6).

Sampling unit/replication	Species	Family	Life form	2004 Mean abundance (%)	2011 Mean abundance (%)	Contribution (%)
Upper SAS/summit aspects	*Kunzea muelleri*	Myrtaceae	Shrub	3.08	3.44	9.38
	*Epacris microphylla*	Ericaceae	Shrub	3.76	4.24	9.03
	*Poa* sp*.*	Poaceae	Graminoid	7.4	9.76	8.33
	*Celmisia costiniana*	Asteraceae	Forb	3.72	3.8	5.69
	*Empodisma minus*	Restionaceae	Graminoid	1.6	2	4.88
	*Phebalium ovatifolium*	Rutaceae	Shrub	1.6	1.52	4.6
	*Grevillea australis*	Proteaceae	Shrub	1.8	1.48	4.59
	*Trisetum spicatum*	Poaceae	Graminoid	2.04	1.88	4.45
	*Prostanthera cuneata*	Lamiaceae	Shrub	1.32	1.32	3.85
	*Microseris lanceolata*	Asteraceae	Forb	1.44	1.44	3.75
Upper SAS/summits	*Kunzea muelleri*	Myrtaceae	Shrub	5.24	5.72	7.13
	*Phebalium ovatifolium*	Rutaceae	Shrub	3.8	3.72	4.88
	*Grevillea australis*	Proteaceae	Shrub	4.28	3.48	4.8
	*Empodisma minus*	Restionaceae	Graminoid	4.6	5.44	4.4
	*Prostanthera cuneata*	Lamiaceae	Shrub	3.2	3.56	4.22
	*Epacris microphylla*	Ericaceae	Shrub	8.32	9.68	4.17
	*Pentachondra pumila*	Ericaceae	Shrub	3.16	2.92	4.08
	*Agrostis* sp*.*	Poaceae	Graminoid	0.04	3.8	4.05
	*Carex breviculmis*	Cyperaceae	Graminoid	0	3.84	4.02
	*Poa* sp*.*	Poaceae	Graminoid	12.64	15.68	3.31
Lower SAS/summit aspects	*Kunzea muelleri*	Myrtaceae	Shrub	2.32	2.72	7.85
	*Poa* sp*.*	Poaceae	Graminoid	7.6	9.92	7.6
	*Celmisia costiniana*	Asteraceae	Forb	4.76	4.6	6.72
	*Epacris microphylla*	Ericaceae	Shrub	3.52	4.08	6.24
	*Phebalium ovatifolium*	Rutaceae	Shrub	2.04	1.8	5.57
	*Empodisma minus*	Restionaceae	Graminoid	1.76	1.8	5.01
	*Grevillea australis*	Proteaceae	Shrub	1.92	1.6	4.5
	*Trisetum spicatum*	Poaceae	Graminoid	2.12	2.2	4.31
	*Pentachondra pumila*	Ericaceae	Shrub	1.4	1.6	3.91
	*Prostanthera cuneata*	Lamiaceae	Shrub	1.12	0.96	3.04
Lower SAS/summits	*Kunzea muelleri*	Myrtaceae	Shrub	4.72	5.28	5.82
	*Phebalium ovatifolium*	Rutaceae	Shrub	4.52	3.96	5.25
	*Empodisma minus*	Restionaceae	Graminoid	4.72	4.32	4.62
	*Grevillea australis*	Proteaceae	Shrub	4.56	3.72	4.21
	*Agrostis* sp*.*	Poaceae	Graminoid	0	3.64	3.76
	*Carex breviculmis*	Cyperaceae	Graminoid	0.12	3.72	3.59
	*Pentachondra pumila*	Ericaceae	Shrub	3.92	4.4	3.51
	*Epacris microphylla*	Ericaceae	Shrub	7.72	9.04	3.02
	*Luzula alpestris*	Juncaceae	Graminoid	0.04	2.92	2.89
	*Acetosella vulgaris*	Polygonaceae	Forb	1.16	3.56	2.87

### Spatial and temporal patterns in functional traits and CTWMs

**Figure 3.**
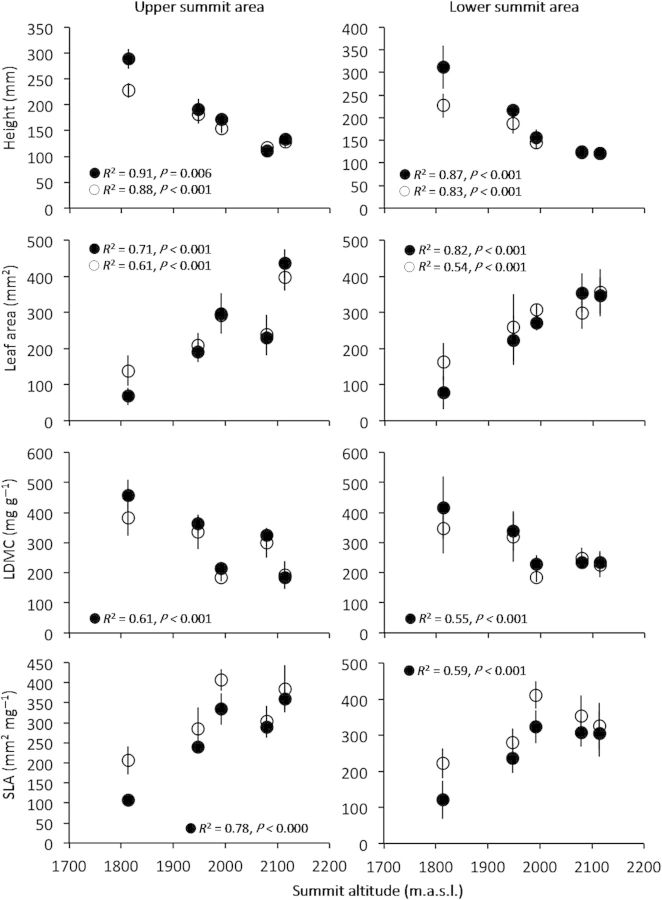
The CTWM ± 95 % confidence intervals for the four functional traits, plant height, leaf area, LDMC and SLA, based on species abundance in the upper and lower SASs. 2004 data = closed circles, 2011 data = open circles. Simple linear relationships (using all site aspect data) between site elevation and CTWM values are only displayed for *R*^2^ ≥ 0.5 and *P* < 0.05.

The most substantial differences in the CTWM occurred at the lowest summit, Clarke 5, of up to 110 % for leaf area and SLA traits between years. At the higher-elevation summits, the CTWM for leaf area and SLA traits increased by up to 26 % in 2011, with the largest increases within the lower SASs (Table 1). The CTWM for SLA increased significantly between 2004 and 2011 in both the upper and lower SASs of Clarke 5 (1813 m) and Clarke 3 (1992 m) (Fig. 3). The CTWM for leaf area was significantly greater at Clarke 5 in the upper SASs between 2004 and 2011 and the CTWM for plant height decreased significantly between 2004 and 2011 at Clarke 5 in the upper and lower SASs (Fig. 3).

Paired *t*-tests between the CTWM for each trait, combining all sites and using summit aspects as replicates (*n* = 20 for each test), revealed several significant differences between years; on the upper summit areas the CTWM for plant height and LDMC were significantly lower in 2011 (*P* = 0.004 and *P* = 0.003, respectively), whereas the CTWM for SLA was significantly higher (*P* < 0.001). On the lower summit areas, the significant differences trended in the same direction (plant height, *P* = 0.019; LDMC, *P* = 0.011; SLA, *P* < 0.001).

Across the gradient of summits there were several significant linear relationships with good predictive power between the CTWM value for each of the measured traits and site elevation. Notably, plant height (decrease with elevation), leaf area (increase with elevation), LDMC (decrease with elevation) and SLA (increase with elevation) were all significantly related to elevation in 2004, whereas only plant height and leaf area were significantly related to elevation in 2011 (Fig. 3). Considering species preferentially organize themselves across the gradient in relation to environmental variables (Pickering and Green 2009; Venn *et al.* 2012*a*), we are confident that any intraspecific trait variation will be overridden by the strong patterns in species distributions and life forms at these sites.

The relationships between site elevation and the CTWM values for each trait (Fig. 3) demonstrate how the CTWM of each site is determined floristically (by abundance) across the gradient of sites. Additionally, the significant increases in the CTWM for SLA between 2004 and 2011 can be attributed to the changes in mean abundance of key shrub species (Table 4) with small, tough leaves such as *Kunzea muelleri* which increased in abundance. The significant decreases in the CTWM for plant height at Clarke 5 (Fig. 3), however, can mostly be attributed to the relative increases of the short/prostrate shrub *Eparcis microphylla* (Ericaceae) on this summit.

## Discussion

### Linking changes in vegetation composition with functional composition

Our exploratory analyses have demonstrated how a short-term change in vegetation composition affects the CTWM and how it varies preferentially across the gradient of summits. Not surprisingly, the patterns in life forms across sites strongly reinforce the patterns in the CTWM, as do broad functional groups of species across other environmental gradients (Reich *et al.* 1997; Choler 2005).

In general, with increasing elevation within both the lower and upper SASs, shrub species appear less abundant and forb species more so, whereas the proportion of graminoid species tends to increase in abundance with elevation, a trend similarly reported in other local studies (Venn and Morgan 2005; Venn 2007). Across the gradient, the CTWM for plant height decreases significantly with elevation as the proportion of tall shrub species (particularly *Phebalium ovatifolium*) decreases with longer snow seasons and lower temperatures at higher elevations. Similarly, the CTWM for the leaf traits corresponds with the higher proportion of forbs and graminoids at the higher elevations; SLA and leaf area increase with elevation and LDMC decreases, reflecting the higher abundances of smaller, compact forb and graminoid species (particularly the sedges *Carex hebes*, *C. breviculmis*) at the higher elevations, as is found in similar studies (Kudo *et al.* 1999; Choler 2005; Venn *et al.* 2012*a*). These trends were also apparent at the scale of SAS within mountains, where directional differences in life form and the CTWM exist between the upper and lower summit areas.

Species with small, thick leaves and low SLA are key features of high, wind-exposed sites (Tranquillini 1964; Kudo *et al.* 1999; Choler 2005), as is the high abundance of short-statured species as found at these sites, but also many of the shrub species dominant at the lower elevations, which is typical of many Australian shrubs (Groves 1994). This is consistent with studies relating canopy architecture and plant height to wind in exposed sites (Caldwell *et al.* 1974; Smith *et al.* 1995; Körner 1999) or possibly phylogenetic conservatism of habitat (Cornwell and Ackerly 2009). In the context of this study, high SLA and leaf areas at the higher, supposedly more stressful sites appear counterintuitive and are inconsistent with similar studies of leaf traits across growing season and snowmelt gradients (Kudo *et al.* 1999; Spasojevic and Suding 2012). In the Snowy Mountains, fewer shrubs are present at higher elevations and forbs at high elevations are generally selected to have smaller leaves, although their leaves are generally fleshy and larger than those of the shrubs, hence the patterns in SLA and leaf area across the gradient. However, at the highest wind-swept areas of feldmark, several tough, dwarf, prostrate shrub species are present (such as *Epacris* and *Chionohebe* spp.) in an environment where long-lived species are selected for. Large-scale interspecific comparisons have strengthened the idea that SLA is related negatively with leaf life span and positively with relative growth rate (Reich *et al.* 1999; Wright *et al.* 2004), a potentially advantageous strategy in harsh climates with short growing seasons. However, a longer snow season at the higher summits in the Snowy Mountains may actually provide more favourable conditions in early spring when plants are protected from early-season frosts and winds, whereas they would be exposed earlier at the lower summits (Inouye *et al.* 2002; Venn *et al.* 2012*b*), which may have caused these unexpected patterns in the CTWM for SLA across the gradient. This is certainly the case around the edges of late-lying snowpatches in the Snowy Mountains, which melt out earlier than the centre of the snowpatch (Venn *et al.* 2011), and where tough, high fibrous (low SLA) species are more abundant in the early-exposed alpine areas (Choler 2005). Conversely, low LDMC and large leaf areas at higher elevations may be indicative of fast resource acquisition and relatively high productivity, even in areas where growing seasons are short and unpredictable (Kudo *et al.* 1999).

Only a few studies have documented temporal functional diversity or functional composition change in plant communities, with mixed results; but see the review in Díaz and Cabido (2001). Here, significant differences in the CTWM of several traits between years, but some non-significant differences in terms of species composition (from ANOSIM analyses) point to species abundance change, rather than high turnover or increases of new species. These changes are likely attributable to the overall large increases in shrub and graminoid abundance across the gradient, which in turn has consequences for driving overall community assembly processes given the trait-space and life-history strategies of these broad life-form groups (Díaz and Cabido 2001; Westoby *et al.* 2002). Although temperatures did not change significantly over the study period, precipitation did. The recent spikes in precipitation, however, are unlikely to have caused the patterns observed in the functional composition in this study; instead these may be realized in the subsequent 1–2 growing seasons as the herbaceous vegetation responds to the additional rainfall after many years of drought conditions.

### Community composition and ecology across the environmental gradient

Our analysis of functional patterns from leaf traits may be used to help describe the mechanisms driving community composition across this elevation gradient of summits (McGill *et al.* 2006). Overall, species have sorted themselves preferentially across the gradient, most likely as a result of abiotic filtering (Venn *et al.* 2011), the harsh conditions at the higher summits compared with the lower summits, and possibly through the additional implications of biotic interactions. Both interaction types are ultimately predetermined by species' inherent functional traits and the functional trait space that each species occupies (Díaz and Cabido 2001). Whereas a significant change in the CTWM across the gradient for a specific trait could indicate a threshold for an abiotic filter, it could also be due in part to biotic filtering, where positive or negative interactions between plants interact with environmental factors preferentially across a gradient to either constrain or facilitate species with certain traits or trait combinations (Schöb *et al.* 2012; Spasojevic and Suding 2012).

Although we did not formally address the issue of intraspecific trait variation in this study, we noted that there is little variation in the traits measured within species across the gradient of sites; rather most of the variation in plant traits across the gradient comes from the variation in species abundances, as demonstrated by the CTWM. Across gradients such as this one, there is an expectation that the CTWM of each trait will be a reflection of a gradient of abiotic stress: higher at the higher-elevation summits and lower at the lower-elevation summits. Conversely, the CTWM for certain traits might reflect some overriding biotic interactions which ameliorate stressful abiotic conditions as per the stress-gradient hypothesis (Bertness and Callaway 1994; Kikvidze *et al.* 2006): in areas where abiotic stress is low and negative biotic interactions (competition) are expected (at the lower-elevation summits: higher temperatures, longer growing seasons) (Callaway *et al.* 2002; Cornwell and Ackerly 2009; Venn *et al.* 2009). This may explain the patterns in two of the four traits considered (the high CTWM values for SLA and leaf area), indicating that biotic filtering likely due to facilitation may be operating at the higher-elevation summits, allowing for relatively high productivity. Further experimental work *in situ* including measures of below-ground plant interactions will be necessary to unravel the interacting effects of biotic and abiotic filtering (Wilson 1993) at these sites. At the lower summits, we observed relatively higher values in the CTWM for plant height and LDMC, strongly driven by the high proportion of shrub and graminoid species. A high proportion of these species can indicate fast and high biomass accumulation, lateral spread and competitive superiority (Westoby *et al.* 2002; Cornelissen *et al.* 2003), as well as leading to increases in soil organic matter and available nitrogen (Myers-Smith *et al.* 2011; Vankoughnett and Grogan 2013). Here, plants may also be less constrained by (relatively) harsh conditions, but biotic filtering such as competition may override other assembly processes.

Trade-offs between an energy-efficient strategy for nutrient conservation, with a high capacity for resource acquisition under harsh conditions, will likely drive future shifts in functional traits and species composition across the gradient (Choler 2005). However, the interaction between abiotic filters (such as growing season length and variability) and biotic filters will ultimately determine whether short-term temporal shifts in species composition will eventually manifest as significant changes to observational trait patterns and ecosystem functioning in the long term. In the short term, the herbaceous species with high SLA and high productivity at high elevations must continue to ‘hold their ground’. However, the relatively tall graminoids and shrubs, with thick, tough leaves (low SLA) that dominate the lower summit areas and at lower elevations more generally, are likely to maintain their dominance, increase in abundance and out-compete other life forms in the local species pool.

## Sources of Funding

Australian Alps Liaison Committee, the New South Wales National Parks and Wildlife Service, the Australian Government and the partners in the National Climate Change Adaptation Research Facility (NCCARF) consortium.

## Contributions by the Authors

S.V. was involved in securing funding, data collection, data analysis and manuscript preparation. C.P. and K.G. were involved in securing funding, data collection and editing the manuscript.

## Conflicts of Interest Statement

None declared.
